# Immersive Media-Based Tourism Emerging Challenge of VR Addiction Among Generation Z

**DOI:** 10.3389/fpubh.2022.833658

**Published:** 2022-07-01

**Authors:** Saba Saneinia, Rongting Zhou, Ali Gholizadeh, Fahad Asmi

**Affiliations:** ^1^School of Public Affairs, University of Science and Technology of China, Hefei, China; ^2^Key Laboratory of Immersive Media Technology (Anhui Xinhua Media Co, Ltd.), Ministry of Culture and Tourism, Hefei, China; ^3^School of Humanities and Social Science, University of Science and Technology of China, Hefei, China; ^4^Sharif University of Technology, Tehran, Iran; ^5^Department of Communication of Science and Technology, University of Science and Technology of China, Hefei, China

**Keywords:** immersive addictive behavior, VR self-efficacy, immersive flow, cognitive behavioral framework, tourism, generation Z

## Abstract

The virtual reality (VR) applications in entertainment and tourism industry have become growingly intense among generation Z. Interestingly, some pilot research on tourism studied concluded the positive impact of its flow experience on adoption of VR tourism, which is also driving the risk of immersive addictive. In the context of tourism and information and communication technology (ICT)-based innovation, there is a lack of immersive addictive behavior (IAB)-related literature. In addition, during the currently ongoing pandemic crisis, VR technology has gained particular importance in the tourism industry among generation Z. The present venture underlines the mechanism of IAB, investigates the VR addiction while underlining the cognitive abilities of individuals. This study applies empirical framework of cognitive–behavioral model. Results demonstrate that in the case of VR tourism, the immersive experience (presence and flow) determines the addictive behavior. Furthermore, VR imagery (VI), psychological curiosity (PC), and VR convenience (VRC) have significant influence on the VR presence and immersive flow. Moreover, the practical and theoretical implications have been discussed in the current research to prevent IAB.

## Introduction

The virtual reality (VR) technology provides users the simulated experience of reality, which offers several applications for the entertainment and tourism industry in recent years (1–3). The VR environment offers a multi-dimensional immersive environment that allows people to observe reality (4, 5) stated that illusions would happen when the sense of placing in a simulated world occurred for users (6) determined that VR headsets offer stereo vision in the virtual environment. Therefore, the users of immersive media can get involved with an amusing environment virtually (7, 8) mentioned that VR can play a significant role in the tourism industry. To underline the dark aspect of VR as technology in the tourism industry, this study underlines that addictive behavior evolved over the basis of cognitive factors. In 2016, Webster defined addiction as a powerful and harmful need to do or have something regularly. VR creates an immersive world based on technology that is so attractive for users and makes them react the way they do in the real world. This attractiveness and the capacity of VR push the users to use it frequently, which leads them to VR addiction (9).

This research proposed the distal factors, including VR immersive presence (IP) and immersive flow (IF). In addition, the proximal cognitive factors include curiosity, convenience, and imagery.

In this research, we consider six hypotheses to evaluate the effects of distal causes, proximal causes, and a moderator on immersive addictive behavior (IAB). The distal causes include VR imagery (VI), psychological curiosity (PC), and VR convenience (VRC); proximal causes including IP and IF, and VR usage frequency (UF), and the moderator in our model.

This study applies the empirical framework of the cognitive–behavioral model, which used a questionnaire survey to collect data and analyze the hypotheses. The pilot study was performed on 30 students. This questionnaire has been given to nearly 1,200 students/users within China. We have gathered 910 responses from the sampling frame and subsequently, for data analysis, 776 respondents' responses have been considered.

## The Addiction of VR Tourism and Emerging Challenge

The VR technology has come up with new perspectives and possibilities, which can be fruitful for the tourism industry to grow and develop. Despite the new trends emerging from the application of this certain technology, still there are various sorts of applications that have been utilized by the tourism industry. Having an insight within these persisting applications in tourism we get enlightened that it is significantly affecting tourism and extending the immense opportunities for the tourism professionals and the researchers (10). To combine media factors in tourism with fewer resources, (11) considered gamified mobile and smart tourism. The virtual conditions simulated in the virtual world, provided by the student's recognition of unique immersion experiences, encourage them to discover the variety of conditions, objects, situations, and methods identified in the pool of literature (12). As suggested by (13), generation Z' tourism activities are altered by ingrained tourism willingness that supports some of the simulated technological environments of the same portable devices. In recent years, some public investment researchers have considered the use of apps for significant purposes in tourism 14 emphasized the use of VR technology to advance VR gaming apps, which is particularly worthy of tourism activities. In addition, gaining a situation to interact with media to promote tourism comes by VR tourism apps conditions (14). Furthermore, the background of addiction to these apps when it comes to motivating visitors within VR tourism must be rated as well.

The title addiction has been obtained from Latin with an accurate definition of giving over, which includes either good or bad aspects (15). Benjamin Rush, a renowned figure of the nineteenth century, depicted that this was a condition of the will, and later (16) introduced addiction as a way beyond one's control. Meanwhile, addiction has been a manifold phenomenon because it is not technologically the result of a substance or program technology. Somehow, it can be observed that they are consequences of the activity and mental structures of humans. On the other hand (17), stated that addiction represents the relationship between beings and goals in their circumstances. Researchers noticed that mobile attractiveness and Internet and technology addiction are rising as mental health concerns (18, 19). Four varieties of knowledge technology addictions with notable characteristics had recognized by (20). Specifically, it covers Internet media disturbance, Internet addiction, Social-Networking addiction, and smartphone addiction. Easy access to mobile phones affects technology addiction among Generation Z (children who become adults in the 2020s), and this addiction can impair academic achievement and cause a sense of hopelessness (18, 21) believed that there is a lack of research on addiction in a media-rich environment. In addition, research of (22) concluded comparable results, which show the importance of our current study.

Immersion media as the challenges of providing a sequence equivalent to tourism and realization in a completely media-rich environment with states of action flow, confusion, and acceptable vision that is as a sense of presence has increased. Worryingly, it can trigger behavioral or addiction mental health diseases. Severe use of the Internet as a type of disorder based on technology or in the name of Internet addiction was registered by the American Psychiatric Association (The Diagnostic and Statistical Manual of Mental Disorders, Fifth Edition (DSM-5)) in 2013 (23). VR tourism apps, in some cases, can negatively affect users and people who interact with them and lead them to addiction (24). The reason for this is that VR creates a computer environment that is very powerful and creates a real-life atmosphere for users. The purpose of this study is to demonstrate the prevailing conditions in IAB in the tourism apps industry and possible suggestions for its defeat.

## Framework (IAB of Cognitive–Behavioral View)

The problematic IT usage has been identified for the cognitive–behavioral framework. The combination of distal and proximal causes revealed as the results. The framework's focus is the individual's cognitions/thoughts, which are the primary basis of abnormal behavior (25, 26). In this framework, the distal causes direct to objects that have an important position in the elaboration of problematic usage, but this is not a natural effect. As mentioned earlier, proximal causes refer to items that affect directly problematic use (25). In the pool of literature, rare studies used to explain IAB, but this paradigm frequently applied to examine the history of the different kinds of addiction behavior, same as VR leisure activity, mobile social networking sites (SNS), online gaming, online gambling, and Internet overuse (24, 27–30). Furthermore, there is a lack of studies in the meaning of tourism with the same assumed modeling; however, some studies considered relevant variable elements. Distal and variably proximal causes have applied to the feasibility of scholastic achievement examined by health results (31). The relationship of VI, PC, and VRC with IP and IF did not discover by no researcher in the previous studies. Therefore, in this study, it is considered that the effectiveness and evaluation of an immersion environment for IAB and tourism are due to the significant impact that this relationship creates. Authors have considered the addictive behavior of immersion as an inner factor. VI means the ability to shoot PC (i.e., behavioral mode) and VRC (i.e., cost), as more distant reasons to consider. Due to the great connection that VI has with emotions, it helps to protect emotional situations and has secondary consequences in trends, behaviors, and immersive media, like VR tourism apps, create a sense of presence in people with the ability to describe up and down (14, 23, 32, 33). The VI, PC, and VRC could have an important impact on the uses of consumers' VR apps (22).

Researchers have proven that all the variables, including VI, PC, and VRC, influence the usage of users on VR games as they cause more attraction (32, 34, 35). However, we have assumed VI, PC, and VRC as distal variables in immersive addiction. Presence as another element is considered as a sensation that is a non-physical concept to show the physical impact of sensation of presence (36). Other scholars supposed presence as an important variable, which leads the emotions and actions of the users toward the virtual environment (37, 38).

The feeling of presence can enhance the empirical intensity of VR gaming, which provides viewers with the impression of being in the game (36). In the tourism context (39), mentioned the effectiveness of VR *via* presence and emotion as a tourism-marketing tool. In addition, the VI experiment was an impact of physical manner in the tourism industry as reported by scholars (40).

Additionally based on the suggestions of other researchers, the interaction achieved through electronic tourism sites creates an image that illuminates the presence's existence like a flow (40, 41). “The holistic sensation that people feel when they act with total involvement.” It is the definition of flow experience based on research of (42). Mental status is defined as the experience of flow with cognitive immersion, misuse of time, and entertainment for users (43). The concept of VR and augmented reality is defined when a person feels that he/she has lost time and space and is fully involved in the content of the IP, which is a psychological element (35). In addition, another research showed that the experience of flow drives IAB (43). In this study, distal causes include VI, PC, and VRC as individuals' imageries and dreams, and states of behavior, in the content of IAB. Additionally, in the framework of cognitive–behavior, proximal causes have been utilized IP and IF in the modeling. The authors in this study tried to classify the motives of why people exhibit IAB.

### Distal Causes

The ability of imagination, which is defined as a mentally generated illustration of an object, event, or feeling, is VI (44). IP and VI have a meaningful relationship, as two theoretical constructs (32). The sense of presence is a decisive factor driving consumers' emotions and behaviors approaching the virtual environment (37). The hypothesis of Rodríguez-Ardura and Meseguer-Artola's research supposed that imagery changes flow positively (45). The essential variables that determine players' feelings in virtual surroundings in this research connect the lack in prior scholars *via* linking VI and IAB with IP and IF.

H1: VR Imagery as Distal Cause is a significant effect on introduced Proximal Causes, which are Immersive feeling of Presence (IP) and Immersive Flow (IF).

Psychological curiosity is specified *via* a preference to indeterminacy that causes answers the same as examining, forming, and investigating in a mental manner (46, 47). The different aspect of user action is curiosity. The VR application designers persuade curiosity *via* performing or augmenting the saliency of data gaps, certain constant connection among member action measures and curiosity, which is one of the most significant difficulties for them (24). In another study, curiosity has been considered as a piece of the feeling of the meaning of presence (22). Furthermore, the researchers in the virtual context highlighted the important straight outcome of the presence on the priority, and PC, i.e., a greater feeling of presence while the experiences of VR direct to greater enthusiasm (40). Accordingly, in our modeling, we supposed there is a link within PC and IF in the content of the VR tourism apps.

H2: Psychological Curiosity as Distal Cause and Immersive sense of Presence (IP) and Immersive Flow (IF) as Proximal Causes have a positive relation.

The problem of how to extract VRC has been an essential academic and industrial subject. The convenience has been noticed by scholars in VR and also tourism context, especially during the COVID-19 pandemic (39, 48). Despite the effects of COVID-19 lockdowns that have not been studied in this research, it is expected that this factor has intensifying influences on IAB. Consumers need to feel completely comfortable. Comfort can provide an effective framework, as reported by (49), to study how he/she interacts with different models of activity. A review by (35) showed how the presence of immersion changes the thinking of ordinary users. He also showed how technical and emotional conveniences could lead to behavioral changes in users' emotions. Furthermore, he illustrates that the primary connection to scholarly convenience correlates to the position of presence and immersion convenience, for example, included cognition and compassion. In addition, Shin had supposed in his study that the Immersive Flow (IF) is related to VR Convenience (VRC), but neither his research nor any other study the relation between IF and VRC is not assessed. As discussed earlier, VR technology involves emotions, such as presence, immersion, interaction, and flow, which are the relationship between comfort and behavior change. In the current research, it has been assumed that with the intervention of IP and IF, VRC would cause IAB.

H3: VR Convenience as distal cause has significant relevance to advanced Immersive Presence (IP) and Immersive Flow (IF) as Proximal Causes.

### Proximal Causes

The passkey to VR effectiveness in various usages is the context of presence (40, 50) determined the presence as a multidimensional understanding, which is arranged within the interplay of multi-sensory data and several cognitive manners (40) determined that flow and feeling of presence, which are the mediators that performed the enthusiasm utilized the similarity of interactivity of VR tourism toward continuation performance.

Furthermore, the researchers studied the user's satisfaction for the useful influence of IP (40). Therefore, we have considered IP as a useful item of IAB, as the addicts obey the happiness of their actions. In the context of VR, the description of the experience of flow in the pool of literature is the impression and mental situation that bodies consider happiness and the senses of time and space will lose meantime, and they do with complete engagement action (35, 42, 51). Among the negative consequences of research-based streaming can be the disorder use of the Internet and addictive behavior (52). As the researchers hypothesize VR tourism application is an effective determinative of the feeling of flow (53), In this study, we hypothesize that IF affects IAB, and also we have covered the details of the VR tourism apps.

H4 and H5: Immersive feeling of Presence (IP) and Immersive Flow (IF) as Proximal Causes are significantly connected to Immersive Addictive Behavior (IAB).

### Moderating Proximal Causes by UF

In VR gaming, UF is an interesting and unique behavioral output aspect in the current mapped out model. The concept of UF requires duplication of situational and particular behavioral ideas; on another hand, UF is an unconscious response to obtain special purposes, which will be continuously renewed (54, 55) described the UF as what technology users are directed to use automatically for tourism. Researchers have suggested that addiction and UF are closely related to information technology research and that UF may diminish users' control skills and then lead them to develop media addiction behavior (56, 57). According to this discussion, authors suppose the UF as a moderator in the modeling for IP and IF; it indicates the rationale of the utilization of experience and sense of the presence of flow in the VR tourism apps, which offers satisfaction, would afford frequency behavior or IAB.

H6: VR Usage Frequency (UF) moderates the associated on among suggested Distal Causes [Immersive sense of Presence (IP) and Immersive Flow (IF)] and Immersive Addictive Behavior (IAB).

## Methodology

This study has applied structural equation modeling to assess the hypothesis in the offered model. Multiple mediations and hierarchal regression methods have been applied to moderate the assessment according to (56) suggested method. In this study, we used a questionnaire survey to collect data and analyze the hypotheses.

### Instrument

We adapted the constructs and the items from the relevant publications to make sure about the validity of the content. The data were collected by doing a questionnaire survey to assess the hypotheses, which are shown in Table 1. For the mental impacts of VR tourism apps, we used five items that reverberate VI (32). PC was focused on using three different items, accentuating the realized curiosity and willingness in VR apps (53). VRC was reflected by using a scale of three items considering control, attributes, and interactivity (43). IP and IF include a scale of three items extracted from (58) and (32) accordingly, IP focused on personal sentiment to describe how the users experience the virtual world, which is IP. IF is the other observation that VR users have experienced, and it is introduced as flow. The scale of three items for UF was extracted from the research of 59. Relevant items of UF discuss the priority of users to apply the VR apps gadget to play. And IAB was tested by scholars for user acts in VR apps processes (54). For each construct, a Likert scale (1: highly disagree and 5: highly agree) has been employed to measure the present model.

**Table 1:** Constructs, instruments, and sources.

**Construct**	**Code**	**Items description**	**Source**
VR Imagery (VI)	VI1	VR tourism apps made me fantasize about having the opportunity to experience it.	(32)
	VI2	It was easy for me to imagine being the part of that VR tourism app.	
	VI3	The mental images that came to mind formed a series of events in my mind in which I was a part of it.	
	VI4	I could easily construct a story about myself and the VR experience based on the mental images that came to mind.	
	VI5	It was convenient for me to imagine being the part of that VR tourism app.	
Psychological Curiosity (PC)	PC1	Using VR tourism apps excites my curiosity	(59)
	PC2	Using VR tourism apps makes me curious.	
	PC3	Using VR tourism apps arouses my imagination.	
VR Convenience (VRC)	VRC1	I feel that I have a lot of control over the content of the tourism sites.	(58)
	VRC2	I use VR tourism apps in an interactive way.	
	VRC3	I feel I can control my visual perspective.	
Immersive Presence (IP)	IP1	The VR Tourism apps create a new world for me, and the world suddenly disappeared when I finished VR experience.	(32)
	IP2	The world generated by VR tourism apps seemed to me like “something I experienced” rather than “something I watch.”	
	IP3	While I use VR, my body stays in the room, but my mind's inside the world created by VR tourism app.	
Immersive Flow (IF)	IF1	While using VR tourism apps, I usually totally focused on the tourism sites.	(58)
	IF2	While using VR tourism apps, I used to be deeply engrossed in the tourism world.	
	IF3	While playing VR games, I used to be absorb intensely.	
Immersive Addictive Behavior (IAB)	IAB1	Using VR tourism apps sometimes interferes with other things	(30)
	IAB2	When I use VR tourism apps, I often feel agitated.	
	IAB3	I have made unsuccessful attempts to reduce the time using VR gadget to use tourism sites.	
Usage Frequency (UF)	UF1	Using VR gadget has become a habit.	(60)
	UF2	I use the new VR gadgets automatically	
	UF3	When I use VR tourism apps, I prefer to use VR gadgets to enjoy it.	
	UF4	While using VR tourism apps, I adopt to new VR gadgets easily.	

### Sampling/Data Collection

In this section, three subsections are made for the tools suggested. The first part proposes the aim of this study. The second part dealt with demographic issues. The third section includes items based on Likert scale structures. During data collection, several items were entered with a reverse code, which is used to worry about quality, and in the analysis, those respondents who were hidden were removed. In this study, we measured students/users who are VR tourism apps users of different age groups.

To remark on the intercommunicating issues and lexical meaningfulness, a pilot study was accomplished on 30 students. This survey has been adapted according to the report of (https://www.wjx.cn), which is an online questionnaire platform.

We have created and passed QR codes and the Weblink on WeChat and some other social media platforms *via* the social media groups of students in different schools to get more survey responses. The study survey was conducted from April to September of 2019. For sampling, the initial research intentionally aimed at users in Fuyang, Nanjing, Wuhan Shanghai, Shenzhen, and Hefei by adopting convenience sampling.

Furthermore, while the other half of the random sampling perceived, research users' responders came from almost different areas in China who are VR tourism apps users. This questionnaire has been given to nearly 1,200 students/users within China. We have gathered 910 responses from the sampling frame. However, owing to incomplete, haphazard, and not satisfactory responses, 134 questionnaires have been excluded from the overall respondents.

Subsequently for data analysis, 776 respondents' responses have been considered appropriate, which covers the criteria stated by (61) when the population is unknown and researchers lack the exact number. Consequently, the first and the last halves of respondents were compared to figure out the non-response bias in the collected data. Perhaps it was observed that the data have not been influenced by the non-response bias. Correspondingly, the demographics distribution of the data unveiled that 76.20% of the present sample have been male ranging from the age group of 25 to 35 years. Meanwhile, 40% of the respondents reflected that initiated to get involved in the VR tourism apps as depicted in Table 2.

**Table 2:** Surveyed sample profile.

**Characteristic**	**Detail**	**Frequency**	**Percentage**
Gender	Male Female	591 185	76.20 23.80
Age	Under 25 25-35 Above 35	124 547 105	15.98 70.49 13.53
Using VR tourism apps for last	<1 month 1 to 3 months 3 to 5 months More than 5 months	308 329 126 13	39.70 42.40 16.20 01.70

As mentioned above, the exclusion criteria include incomplete, haphazard, and not satisfactory responses. For example, responses that were collected from those students who never used VR tourism apps or those responses who used other apps, which are not in the VR tourism app categories, were excluded.

## Analysis

The following segment can be categorized into 3 parts that have been arranged to dig out the reliability, the validity of the present model, to seek out this concerning portion structural analysis has been applied. In addition to this total effect, moderation and mediation have also been reflected within this segment.

### Measurement Model

To estimate the reliability and significance of all cover models and structures, exploratory case analysis has been used. Convergent validity and reliability preparation factor analysis have been applied for every item. Besides, combined reliability, average variance extracted (AVE), and Cronbach's alpha (α) extracted for each construct measured are shown in Table 3. In addition, Table 3 shows the convergent reliability results.

**Table 3:** Exploring factors and reliability analysis.

**Construct**	**Items**	**λ**	**A**	**CR**	**AVE**
VR Imagery (VI)	VI1	0.860	0.894	0.895	0.631
	VI2	0.839			
	VI3	0.787			
	VI4	0.771			
	VI5	0.706			
Psychological Curiosity (PC)	PC1	0.814	0.777	0.844	0.643
	PC2	0.797			
	PC3	0.796			
VR Convenience (VRC)	VRC1	0.886	0.898	0.899	0.748
	VRC2	0.877			
	VRC3	0.831			
Immersive Presence (IP)	IP1	0.856	0.903	0.859	0.672
	IP2	0.855			
	IP3	0.744			
Immersive Flow (IF)	IF1	0.838	0.876	0.858	0.669
	IF2	0.818			
	IF3	0.779			
Immersive addictive behavior (IAB)	IAB1	0.881	0.863	0.897	0.745
	IAB2	0.877			
	IAB3	0.832			
Usage Frequency (UF)	UF1	0.963	0.949	0.961	0.863
	UF2	0.930			
	UF3	0.918			
	UF4	00.903			

The result proved that modules are internally stable and valid. Furthermore, the validity of the discriminant was analyzed by comparing the AVE's square root and internal construct correlation (62). Based on the basic principles of the inference, the edge of the AVE's square root has been the correlation of every concerned construct with the other respective constructs within this mapped out model. Multicollinearity outcomes have been examined and put forward by computing the variance inflation factor (VIF) for the different items of the current construct. The carved out VIF scores have been surprising ranging from 1.031 to 1.5; consequently, all of these scores have been lower than the cut-off value of 10, which has been proclaimed and suggested by (63). Consequently, in this survey, multicollinearity was not a dependency.

In this questionnaire, a single factor recorded was 31.17%, which is the highest variance observed; it reflects that total variance in the present model has not been dominated by a particular construct; perhaps in the underlying model, the conventional methods of bias was not fruitful. Somehow, the fitness criteria of this model have been portrayed through the SEM. The chi-square value was 608.497, whereas the 155 have been the degree of freedom of this mapped out model respectively. In addition to this, fitness indices of the current venture have been observed in the particular range of the suggested cut-off values that (64) indicated earlier.

### Proposed Model

**Figure 1:**
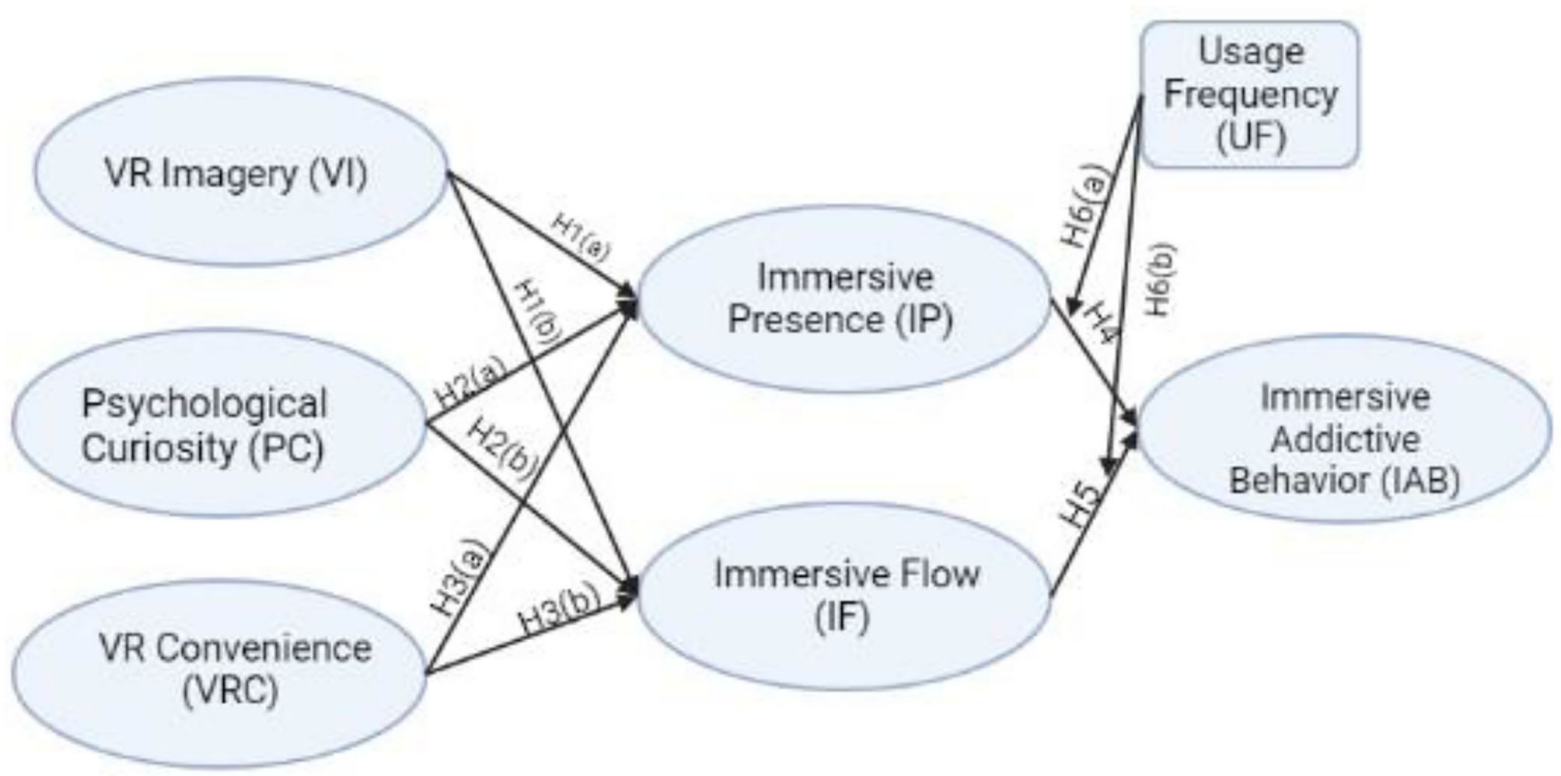
Proposed model of the study.

The standardized assessments are recorded where the graphical description of structural modeling is shown in Figure 1, drawn from the AMOS V.24. Furthermore, to measure the VR tourism apps trends among the users, the control variable appeared with a significant effect. The endogenous contracts of IF, IP, and IAB (R2) reflected the variances of 0.29, 0.34, and 0.30, respectively. Particularly, the different model fit indices that have been investigated reflected the satisfactory range. The study opines through its findings that the age, gender, and education are key factors while knowing that students/users have been impressed by the origin of the tourism apps. The outcomes reflected that VI has a rigorous impact on the IF (H1b: β = 0.52, *p* ≤ 0.001) and the IP (H1a: β = 0.33, *p* ≤ 0.001). As a result of this, H1 (a) and (b) has been accepted. It predicted that the more VI present within the VR addicts can alter their IAB. Similarly, those students/users who pose the strong VI can be addicted to the VR tourism apps (65, 66). In addition to this, the present model digs out that VI has appeared the strongest determinant to elaborate the IF and IP among the VR tourism apps users. The research concluded that great mental dependency, feeling, and interest of VI amongst users could play a significant role in users' IAB. Statistically, PC was seen to have a positive connection with IP (H2a: β = 0.10, *p* ≤ 0.001), and IF was noticed to be non-significant (H2b: β = 0.02, *p* ≥ 0.05). The VRC among users has a positive effect on users' IP (H3a: β = 0.105, *p* ≤ 0.05) and IF (H3b: β = 0.09, *p* ≤ 0.001) as the quantitative results shown in Table 4.

**Table 4:** Parameter estimation for proposed model.

**Sr**.	**Description**	**Beta (β)**	**Significance**	**Result**
H1(a)	VI → IP	0.33	≤ 0.001	Supported
H1(b)	VI → IF	0.52	≤ 0.001	Supported
H2(a)	PC → IP	0.10	≤ 0.05	Supported
H2(b)	PC → IF	0.02	≥0.05	Not Supported
H3(a)	VRC → IP	0.105	≤ 0.05	Supported
H3(b)	VRC → IF	0.09	≤ 0.05	Supported
H4	IP → IAB	0.43	≤ 0.001	Supported
H5	IF → IAB	0.25	≤ 0.001	Supported
H6(a)	IP*UF → IAB	0.10	≤ 0.01	Supported
H6(b)	IF*UF → IAB	0.022	≥0.05	Not Supported

Nevertheless, the smaller impact than the rest of the suggested concerns belongs to VRC. VRC was an effective item in the user's IAB. Such as, (43) illustrated that VRC was a serious item for users' moods to feel the handling of the apps. Whenever determining users' IAB through IP (H4: β = 0.43, *p* ≤ 0.05) and by IF (H5: β = 0.25, *p* ≤ 0.05), the outcomes concluded that IP utilized a more significant impact on users' IAB than their IF. IP boosts people to act in an addictive form, but users' IF will be impressed by focus (43).

### Mediation and Moderation Analysis

We have used the approach suggested by (56), to estimate the mediation result of IP and IF, as declared in H6 (a) and (b), sequentially. Furthermore, to calculate asymmetric confidence intervals (CIs), the 5,000 respondents were included through the sampling method of the bootstrapping. Somehow, the framed hypotheses of H6 have been mediated because the mapped out CIS appeared zero within the lower and higher caps. In addition to this, the research of (67) was employed to calculate the mediation effect. The study put forward that the IF and the IP have shown a strong mediation effect in the users while elucidating the IAB. The outcomes have been adjustable and compatible concerning the output adopted by the unique method of bootstrapping. Moderatos have been measured through the UF of the users by the hierarchical regression analysis. It was articulated that higher UF has increased the association within the user's IP and relatively IAB (H6a: β = 0.10, *p* ≤ 0.001). UF can be a vital factor that alters the experiences of users regarding the VR tourism apps, which was measured as IAB. On the contrary, the IF shows that UF does not have any vital role to explain the H6a. As users are inspired to experience and use the VR tourism apps and feel to find a new environment, the authors suggested that the intentional participation can be most efficient.

## Discussion

The important point of this research is to express the performance of users addicted to VR tourism apps and the desirability and literary depth of the subject. Methods, such as secondary data and interviews, were used alongside studies on VR disease, VR panic, Internet gambling, and technology addiction. In addition to examining empirical data, this research is a summary that can properly address the behavioral consequences of VR tourism apps on users using a multifunctional approach. Another feature of this study is the invention of a unique approach to the formation of cognitive–behavioral changes in users. The behavioral model of VR tourism apps addiction considering VR tourism apps has not been observed in other studies. As users' illness with VR videos has been confirmed in a previous study (18, 68) also confirmed the addictive role of overuse of technology. Researchers have used the VR imaging techniques of (23) with psychological content for behavioral purpose concepts (43, 69). Tourism apps curiosity modifies the sequence of use intention as a convenience factor presented by (70). In the subject of VR tourism, 33 focused on terms of mental imagery, so since no study has ever used addictive conditions in this topic, the authors believed this is an innovation in this research. As previously explained in detail, in this research, we consider six hypotheses to evaluate the effects of distal causes, proximal causes, and a moderator on IAB. The distal causes include VI, PC, and VRC; Proximal causes include IP and IF, and VR UF, and the moderator in our model. In addition, the results are discussed in detail in later sections.

Moreover, the interceding of VI with IF was found out by scholars. In this study model, the consequences of PC on IF are considered insignificant. However, the considerable relation between curiosity and tourism applications has been proven by researchers (24, 70). As the current research suggested the critical role of VI while demonstrating IP and IF, this research centralized on the VR apps-changing influence in the state of VR tourism base, by considering the critical role of VI while illustrating IP and IF, which is also realized as serious being the storage of literature. A reference for study in the academic areas for more than one decade is digital technology, known as a part of Generation Z's lives, which is concerned by (71). By providing a variety of application-based VR tourism (40), examined students' attendance experiences, emotions, and the impact of interest. In addition, in the comparison between IP and IF, we found that IP is more considerable on IAB. The result of IP mixture with UF is making a significant point on IAB, but there is no point for IAB if it composes with IF, yet it shows the effect of relation without UF. The fact that psychological construct with the four knowledge level analysis is guiding the leading to performing addictive behavior has been noticed by this research authors. A serious rate of IAB by the guidance of a high level of VI has been discovered. In addition, the more survey potential of VI and PC in additional studies was recommended by the scholars of this research. Addictions and disorders in technology and VR tourism application caused severe anxieties, which were noticed by researchers. Attending mental health sicknesses, such as addiction, specifically for Generation Z, has been accepted as VR tourism apps and mobile apps result (18, 72) established that the mental skills of students and even parents' job issues could be affected by media apps addiction. For highlighting addiction in the current research content, IP was noticed to be a crucial creation. Therefore, IP, in the tourism context, is an actual obstacle for creating immersive VR tourism environment successes, efficient, and sufficient, which achieves and maintains in this study (45) noticed a related involvement in their studies. In another aspect, the positive role of VR technology in tourism brand manufacturing, which detailed clarification, telepresence, and interactivity, is considered to a higher amount than the normal by researchers (32). VR tourism apps can also increase cognitive potency in tourism content (32, 73, 74) considered, “VR is an immersive, multi-sensational experience.” The correlation between technology development and financial activities has clarified the overall performance of VR in tourism, healing, entertainment, and business, and research areas. Therefore, one of VR apps functions can be a positive aspect of the cost-effectiveness of the country. By the progression of technology in VR apps, the demand for the spirit of presence and flow action be improved; hence, the feeling of engagement and immersive during the use of tourism apps increased. The authors of the current study suggest the fulfillment of the gap, which they detected as any study based on IAB in future research. Additionally, the developers can afford apps in the tourism industry with the help of psychologists to reduce IAB as a result; the students of various age groups can manage it adequately. In addition, people's tastes can be obtained with procedures targeting the methods of VR by the government.

## Conclusion

To monitor the teenager's understanding of IAB, this research used the cognitive–behavioral theory. In addition, we have proven that the “VI” item becomes the greatest power to cause the addiction. Likewise, “flow experience” and “sense of presence” are shown to have the meaningful decisive capability to define IAB. Therewith, balancing assistance at various levels requires to be standardized to limit IAB. With the help of managers, policymakers, and developers, the attractiveness of VR tourism applications can be used instead of creating disorder and addiction to become useful and positive results for Generation Z. This study also has useful results on studies about academic performance and mental health disorders. Furthermore, its meaning can be more modified in the base of VR tourism and tourism industry conditions. Our investigation has the next restrictions, which could guide future studies: (1) first, the study only appropriated the cognitive–behavioral method, which could be more widespread and improved with the other variants of the behavioral and enduring effective conceptual principles to determine behavior of Generation Z; (2) this research concentrated on IAB for youth, which is in Generation Z, which for future research can be extended to other generations and manufacturers using cognitive–behavioral methodologies; (3) because this study used a quantitative method, qualitative methods can also be used to examine and collect data; and (4) furthermore, to make the view of VR addiction more common, other generations can continue to collect data. In addition, future studies can evaluate the effects of COVID-19 and the lockdown on IAB.

## Data Availability Statement

The original contributions presented in the study are included in the article/supplementary material, further inquiries can be directed to the corresponding author.

## Ethics Statement

Ethical review and approval was not required for the study on human participants in accordance with the local legislation and institutional requirements. Written informed consent for participation was not required for this study in accordance with the national legislation and the institutional requirements.

## Author Contributions

SS has completed the analysis results and findings of this research. AG has participated in the introduction and literature of this research. RZ has supervised conducting this research. FA has contributed in data collection. All authors contributed to the article and approved the submitted version.

## Funding

The research funded by the (1) National Social Science Fund of China (Grant ID: 17BXW034); (2) Key Laboratory of Immersive Media Technology (Anhui Xinhua Media Co, Ltd.), Ministry of Culture and Tourism, Hefei, China.

## Conflict of Interest

The authors declare that the research was conducted in the absence of any commercial or financial relationships that could be construed as a potential conflict of interest.

## Publisher's Note

All claims expressed in this article are solely those of the authors and do not necessarily represent those of their affiliated organizations, or those of the publisher, the editors and the reviewers. Any product that may be evaluated in this article, or claim that may be made by its manufacturer, is not guaranteed or endorsed by the publisher.

## References

[1] Williams P Hobson JP. Virtual reality and tourism: fact or fantasy? Tour. Manag. (1995) 16:423–7. 10.1016/0261-5177(95)00050-X

[2] Vince J. Introduction to Virtual Reality. New York: Springer (2004)

[3] Gutiérrez M Vexo F Thalmann D. Stepping Into Virtual Reality. London: Springer. (2008). 10.1007/978-1-84800-117-6

[4] Fernández-palacios BJ Morabito D Remondino F. Access to complex reality-based 3D models using virtual reality solutions. J. Cult. Herit. (2017) 23:40–48. 10.1016/j.culher.2016.09.003

[5] Slater M. Place illusion and plausibility can lead to realistic behaviour in immersive virtual environments. Philos Trans R Soc B Biol Sci. (2009) 364:3549–57. 10.1098/rstb.2009.0138 19884149 PMC2781884

[6] Prasuethsut L. HTC Vive: Everything You Need to Know About the SteamVR Headset. (2017).

[7] Chambel T. Interactive and Immersive Media Experiences. in *Webmedia'*16, 22nd. Brazilian Symposium on Multimedia and the Web 2984746. (2016).

[8] Saneinia S Gholizadeh A Zhou R. A holistic view of tourism development and potential policy concerns: a case of Birjand City, Iran. African J Hosp Tour Leis. (2020) 9:1–7.

[9] Rajan AV Nassiri N Akre V Ravikumar R Nabeel A Buti M. Virtual reality gaming addiction. In: 2018 Fifth HCT Information Technology Trends (ITT). IEEE (2018), pp. 358–63. 10.1109/CTIT.2018.8649547

[10] Guttentag DA. Virtual reality: Applications and implications for tourism. Tour Manag. (2010) 31:637–51. 10.1016/j.tourman.2009.07.00335069015

[11] Garcia A Linaza MT Gutierrez A Garcia E. Gamified mobile experiences: smart technologies for tourism destinations. Tourism Rev. (2019) 74:30–49. 10.1108/TR-08-2017-0131

[12] Bhattacharjee D Paul A Kim JH Karthigaikumar P. An immersive learning model using evolutionary learning. Comput Electr Eng. (2018) 65:236–49.

[13] Robinson VM Schänzel HA. A tourism inflex: generation Z travel. Experiences. (2019)5:127–41. 10.1108/JTF-01-2019-0014

[14] Marchiori E Niforatos E Preto L. Measuring the media effects of a tourism-related virtual reality experience using biophysical data. In: Information and Communication Technologies in Tourism 2017. Cham: Springer (2017). p. 203–15. 10.1007/978-3-319-51168-9_15

[15] David D Arman E Chandra N Nadia N. Science direct development of escape room game using VR technology. Procedia Comput Sci. (2019) 157:646–52. 10.1016/j.procs.2019.08.223

[16] Heather N. A conceptual framework for explaining drug addiction. J Psychopharmacol. (1998) 12:3–7. 10.1177/026988119801200101 9584962

[17] Weil AT. Drug, Set, and Setting: The Basis for Controlled Intoxicant Use by Norman Zinberg. New Haven, CT: Yale University Press (1984).

[18] Jamir L Duggal M Nehra R Singh P Grover S. Epidemiology of technology addiction among school students in rural India. Asian J. Psychiatr. (2019) 40:30–38. 10.1016/j.ajp.2019.01.009 30716701

[19] Bhardwaj MAM. Mobile phone addiction and loneliness among teenagers. Int J Indian Psychol. (2015) 2:27–34. 10.25215/0203.062

[20] Sigerson L., Li, AYL, Cheung MWL, Cheng C. Examining common information technology addictions and their relationships with non-technology-related addictions. Comput. Human Behav. (2017) 75, 520–526. 10.1016/j.chb.2017.05.041

[21] van Herpen E van den Broek E van Trijp HC Yu T. Can a virtual supermarket bring realism into the lab? Comparing shopping behavior using virtual and pictorial store representations to behavior in a physical store. Appetite. (2016) 107:196–207. 10.1016/j.appet.2016.07.033 27474194

[22] Suh A Prophet J. Computers in human behavior the state of immersive technology research: a literature analysis. Comput Human Behav. (2018) 86:77–90. 10.1016/j.chb.2018.04.019

[23] Ji JL Kavanagh DJ Holmes EA MacLeod C Di Simplicio M. Mental imagery in psychiatry: conceptual and clinical implications. CNS Spectr. (2019) 24:114–126. 10.1017/S1092852918001487 30688194

[24] Jeng MY Pai FY Yeh TM. The virtual reality leisure activities experience on elderly people. Appl Res Qual Life. (2017) 12:49–65. 10.1007/s11482-016-9452-0

[25] Maier C Laumer S Weinert C Weitzel T. The effects of technostress and switching stress on discontinued use of social networking services: a study of Facebook use. Infm Syst J. (2015) 25:275–308. 10.1111/isj.12068

[26] Davis R. A cognitive-behavioral model of pathological Internet use. Comput Human Behav. (2001) 17:187–95. 10.1016/S0747-5632(00)00041-8

[27] Wang HY Sigerson L Cheng C. Digital nativity and information technology addiction: age cohort versus individual difference approaches. Comput. Human Behav. (2019) 90:1–9. 10.1016/j.chb.2018.08.031

[28] Mancini T Imperato C Sibilla F. Does avatar's character and emotional bond expose to gaming addiction? two studies on virtual self-discrepancy, avatar identification and gaming addiction in massively multiplayer online role-playing game players. Comput. Human Behav. (2018) 92:297–305. 10.1016/j.chb.2018.11.007

[29] Zhang R Bai B Jiang S Yang S Zhou Q. Computers in human behavior parenting styles and internet addiction in Chinese adolescents: conscientiousness as a mediator and teacher support as a moderator? Comput. Human Behav. (2019) 101:144–150. 10.1016/j.chb.2019.07.019

[30] Gong M Yu L Luqman A. Understanding the formation mechanism of mobile social networking site addiction: evidence from WeChat users. Behav Inf Technol. (2019) 0:1–16. 10.1080/0144929X.2019.1653993

[31] Schafer MH Ferraro KF. Social science and medicine distal and variably proximal causes: education, obesity, and health. Soc Sci Med. (2011) 73:1340–8. 10.1016/j.socscimed.2011.08.010 21920651

[32] Bogicevic V Seo S Kandampully JA Liu SQ Rudd NA. Virtual reality presence as a preamble of tourism experience: The role of mental imagery. Tour Manag. (2019) 74:55–64. 009 10.1016/j.tourman.2019.02.009

[33] Zhai X Wang M Zhou R Anwar MA Saneinia S Ahmad I. The nexus between VR affordability, cognition and VR addiction: a gaming perspective. In: 2020 6th International Conference of the Immersive Learning Research Network (iLRN). IEEE (2020). p. 116–124.

[34] Kim Y Lee S. Computers in Human behavior mobile gamer' s epistemic curiosity affecting continuous play intention. focused on players' switching costs and epistemic curiosity. Comput Human Behav. (2017) 77:32–46.

[35] Shin D. Telematics and Informatics The role of affordance in the experience of virtual reality learning: technological and affective affordances in virtual reality. Telemat Informatics. (2017) 34:1826–36. 10.1016/j.tele.2017.05.013

[36] Steuer J. Defining virtual reality: dimensions determining telepresence. J Commun. (1992) 42:73–93. 10.1111/j.1460-2466.1992.tb00812.x

[37] Animesh A Pinsonneault A Yang SB Oh W. An odyssey into virtual worlds: Exploring the impacts of technological and spatial environments on intention to purchase virtual products. MIS Q Manag Inf Syst. (2011) 35:789–810. 10.2307/23042809

[38] Dubosc C Gorisse G Christmann O Fleury S Poinsot K Richir S. Impact of avatar facial anthropomorphism on body ownership, attractiveness and social presence in collaborative tasks in immersive virtual environments. Comput. Graph. (2021) 101:82–92. 10.1016/j.cag.2021.08.011

[39] Yung R Khoo-Lattimore C Potter LE. VR the world: Experimenting with emotion and presence for tourism marketing. J. Hosp Tour Manag. (2021) 46:160–171. 10.1016/j.jhtm.2020.11.009

[40] Tussyadiah IP Wang D Jung TH Claudia M. Virtual reality, presence, and attitude change: empirical evidence from tourism. Tour Manag. (2018) 66:140–154. 10.1016/j.tourman.2017.12.003

[41] Xu F Tian F Buhalis D Weber J. Marketing tourism via electronic games: Understanding the motivation of tourist players. 2013 5th Int. Conf. Games Virtual Worlds Serious Appl. VS-GAMES 2013. (2013). 10.1109/VS-GAMES.2013.6624235

[42] Csikszentmihalyi M. (1975). Beyond Boredom and Anxiety. San Francisco: Josseybass Well-Being Found.

[43] Kim D Ko YJ. The impact of virtual reality (VR) technology on sport spectators' flow experience and satisfaction. Comput Human Behav. (2019) 93:346–56. 10.1016/j.chb.2018.12.040

[44] Pearson J Naselaris T Holmes EA Kosslyn SM. Mental imagery: functional mechanisms and clinical applications. Trends Cogn Sci. (2015) 19:590–602. 10.1016/j.tics.2015.08.003 26412097 PMC4595480

[45] Rodríguez-Ardura I Meseguer-Artola A. E-learning continuance: The impact of interactivity and the mediating role of imagery, presence and flow. Inf Manag. (2016) 53:504–16. 10.1016/j.im.2015.11.005

[46] Nissen M Staunæs D Bank M. A “post-psychological” curiosity about subjectivities and standards. Theory Psychol. (2016) 26:137–43. 10.1177/0959354316628691

[47] McReynolds Paul S Acker Mary PC. Relation of Object Curiosity to Psychological Adjustment in Children. Wiley Soc Res.Child Dev. (2016) 32:393–400 10.1111/j.1467-8624.1961.tb05037.x

[48] Schiopu AF Hornoiu RI Padurean MA Nica A. Virus tinged? Exploring the facets of virtual reality use in tourism as a result of the COVID-19 pandemic. Telemat Informatics. (2021) 60:101575. 10.1016/j.tele.2021.101575 PMC9758450 36569994

[49] Nelson SB Jarrahi MH Thomson L. Mobility of knowledge work and affordances of digital technologies. Int J Inform Manag. (2017) 37:54–62. 10.1016/j.ijinfomgt.2016.11.008

[50] Diemer J Alpers GW Peperkorn HM Shiban Y Mühlberger A. The impact of perception and presence on emotional reactions: A review of research in virtual reality. Front Psychol. (2015) 6. 10.3389/fpsyg.2015.00026 PMC4311610 25688218

[51] Ebert DS Shaw CD. Minimally immersive flow visualization. IEEE Trans Vis Comput Graph. (2001) 7:343–50. 10.1109/2945.965348

[52] Chou TJ Ting CC. The Role of flow experience in cyber-game addiction. Cyberpsychology Behav. (2003) 6:663–75. 10.1089/109493103322725469 14756934

[53] Wei W Qi R Zhang L. Effects of virtual reality on theme park visitors' experience and behaviors: a presence perspective. Tour. Manag. (2019) 71:282–293. 10.1016/j.tourman.2018.10.024

[54] Turel O Serenko A. The benefits and dangers of enjoyment with social networking websites. Eur J Inf Syst. (2012) 21:512–28. 10.1057/ejis.2012.1

[55] Limayem M Hirt SG Cheung CM. How habit limits the predictive power of intention: The case of information systems continuance. MIS Q. (2007) 705–37. 10.2307/25148817

[56] Preacher K Hayes A. Asymptotic and resampling strategies for assessing and comparing indirect effects in multiple mediator models. Behav Res Methods. (2008) 40:879. 18697684 10.3758/brm.40.3.879

[57] Cover R. Online selves: Digital addiction. In: Digital Identities. (2016). 10.1016/B978-0-12-420083-8.00007-9

[58] Kim Y Lee S. Computers in Human behavior mobile gamer' s epistemic curiosity affecting continuous play intention. focused on players' switching costs and epistemic curiosity. Comput Human Behav. (2017) 77:32–46.

[59] Lee CH Chiang H. Sen, Hsiao KL. What drives stickiness in location-based AR games? an examination of flow and satisfaction. Telemat Informatics. (2018) 35:1958–1970. 10.1016/j.tele.2018.06.008

[60] Pillet J Daniel K Carillo A. Email-free collaboration: an exploratory study on the formation of new work habits among knowledge workers. Int J Inf Manage. (2016) 36, 113–125. 10.1016/j.ijinfomgt.2015.11.001

[61] Godden B. Sample size formulas. J Statist. (2004) 3:66.

[62] Fornell C Larcker D. Evaluating structural equation models with unobservable variables and measurement error. J Mark Res. (1981) 18:39–50. 10.1177/002224378101800104

[63] Hair JF Black WC Babin BJ Anderson RE Tatham RL. Multivariate Data Analysis. 7th ed. Essex Pearson Education Limited Harlow (2014).

[64] Hu L Bentler PM. Cutoff criteria for fit indexes in covariance structure analysis: Conventional criteria versus new alternatives. Struct Equ Model A Multidiscip J. (1999) 6:1–55. 10.1080/10705519909540118

[65] Laroche M Bergeron J Barbaro-Forleo G. Targeting consumers who are willing to pay more for environmentally friendly products. J Consum Market. (2001) 18:503–20. 10.1108/EUM000000000615516864889

[66] Arli D Leo C Tjiptono F. Investigating the impact of guilt and shame proneness on consumer ethics: A cross national study. Int J Consum Stud. (2016) 40:2–13. 10.1111/ijcs.12183

[67] Baron RM Kenny DA. The moderator–mediator variable distinction in social psychological research: Conceptual, strategic, and statistical considerations. J Personal Soc Psychol. (1986) 51:1173. 10.1037/0022-3514.51.6.1173 3806354

[68] Guna J Geršak G Humar I Song J Drnovšek J. Influence of video content type on users' virtual reality sickness perception and physiological response. Futur Gener Comput Syst. (2019) 91:263–76. 10.1016/j.future.2018.08.049

[69] Wouters P Oostendorp H. Van, Boonekamp R, Spek E. Van Der. The role of game discourse analysis and curiosity in creating engaging and effective serious games by implementing a back story and foreshadowing. Interact. Comput. (2011) 23:329–336. 10.1016/j.intcom.2011.05.001

[70] Serravalle F Ferraris A Vrontis D Thrassou A Christofi M. Augmented reality in the tourism industry: A multi-stakeholder analysis of museums. Tour Manag Perspect. (2019) 32:100549. 10.1016/j.tmp.2019.07.002

[71] Somrak A Humar I Hossain MS Alhamid MF Hossain MA Guna J. Estimating VR Sickness and user experience using different HMD technologies: an evaluation study. Futur. Gener Comput Syst. (2019) 94:302–316. 10.1016/j.future.2018.11.041

[72] Merkx C Nawijn J. Virtual reality tourism experiences: Addiction and isolation. Tour Manag. (2021) 87:104394. 10.1016/j.tourman.2021.104394

[73] Kang H. Impact of VR on impulsive desire for a destination. J Hosp Tour Manag. (2020) 42:244–55. 10.1016/j.jhtm.2020.02.003

[74] Castelvecchi D. Low-cost headsets boost virtual reality's lab appeal. Nature. (2016) 533:153–4. 10.1038/533153a 27172022

