# Corrigendum: Immersive media-based tourism emerging challenge of VR addiction among generation Z

**DOI:** 10.3389/fpubh.2022.1017242

**Published:** 2022-08-24

**Authors:** Saba Saneinia, Rongting Zhou, Ali Gholizadeh, Fahad Asmi

**Affiliations:** ^1^School of Public Affairs, University of Science and Technology of China, Hefei, China; ^2^Key Laboratory of Immersive Media Technology (Anhui Xinhua Media Co, Ltd.), Ministry of Culture and Tourism, Hefei, China; ^3^School of Humanities and Social Science, University of Science and Technology of China, Hefei, China; ^4^Sharif University of Technology, Tehran, Iran; ^5^Department of Communication of Science and Technology, University of Science and Technology of China, Hefei, China

**Keywords:** immersive addictive behavior, VR self-efficacy, immersive flow, cognitive behavioral framework, tourism, generation Z

In the published article, there was an error regarding the affiliations for Saba Saneinia. As well as having affiliation 1, she should also have Key Laboratory of Immersive Media Technology (Anhui Xinhua Media Co, Ltd.), Ministry of Culture and Tourism, Hefei, China.

In the published article, there was an error regarding the affiliations for Rongting Zhou. As well as having affiliations 2, he should also have Key Laboratory of Immersive Media Technology (Anhui Xinhua Media Co, Ltd.), Ministry of Culture and Tourism, Hefei, China.

In the published article, there was an error regarding the affiliations for Fahad Asmi. As well as having affiliations 2, he should also have Key Laboratory of Immersive Media Technology (Anhui Xinhua Media Co, Ltd.), Ministry of Culture and Tourism, Hefei, China.

In the published article, there was an error in the Funding statement. Authors haven't mentioned any funding detail in the recently published manuscript. The correct Funding statement appears below.

## Funding

The research funded by the (1) National Social Science Fund of China (Grant ID: 17BXW034); (2) Key Laboratory of Immersive Media Technology (Anhui Xinhua Media Co, Ltd.), Ministry of Culture and Tourism, Hefei, China.

The authors apologize for this error and state that this does not change the scientific conclusions of the article in any way. The original article has been updated.

## Publisher's note

All claims expressed in this article are solely those of the authors and do not necessarily represent those of their affiliated organizations, or those of the publisher, the editors and the reviewers. Any product that may be evaluated in this article, or claim that may be made by its manufacturer, is not guaranteed or endorsed by the publisher.

